# A Case Report on an Unknown Etiology Disorder of Consciousness Following Extubation Despite Thorough Evaluation

**DOI:** 10.7759/cureus.75513

**Published:** 2024-12-10

**Authors:** Rena Fukura, Yusuke Ishida, Yuri Kaneda, Katsunori Oe

**Affiliations:** 1 Anesthesiology, Showa University School of Medicine, Tokyo, JPN; 2 Anesthesiology, Showa University Fujigaoka Hospital, Yokohama, JPN

**Keywords:** delayed recovery, disorder of consciousness (doc), electroencephalogram, general anesthesia, glasgow coma scale

## Abstract

Postoperative disorders of consciousness can arise from various factors, making diagnosis challenging. This report presents a case of a disorder of consciousness that occurred after awakening from general anesthesia, in which various examinations including computed tomography (CT) and magnetic resonance imaging (MRI) were conducted, but the cause could not be identified. A man in his 60s with a history of hypertension and cerebral hemorrhage underwent revision total hip arthroplasty under general anesthesia for a left periprosthetic hip fracture. The induction and maintenance of anesthesia were uneventful. Postoperatively, anesthesia was discontinued, and the patient responded to verbal commands, opened his eyes, and was able to follow handgrip instructions, allowing for extubation. However, after moving to the recovery room, his level of consciousness decreased, reaching a Glasgow Coma Scale score of E3V1M3-4. Suspecting prolonged effects of the anesthetic agents, his condition was observed in the recovery room for approximately 30 minutes, but there was no change in his level of consciousness. CT and MRI of the head were performed, but no significant abnormalities were found. Electroencephalogram monitoring did not show any clear epileptiform activity. Gradual improvement in his level of consciousness was noted 60-120 minutes after the initial decline. Subsequently, there were no further episodes of disordered consciousness. Perioperative disorders of consciousness are challenging to diagnose due to the potential effects of anesthetic agents. Although the cause could not be identified in this case, the patient fortunately recovered consciousness without any significant sequelae.

## Introduction

A disorder of consciousness (DOC) is an abnormal state of consciousness due to brain dysfunction. It is characterized by reduced levels of arousal and awareness, and it can be classified into coma, persistent disordered consciousness, and minimally conscious state [1]. Diagnosing DOCs is challenging because they can be caused by various factors. It is crucial to promptly diagnose the cause and initiate appropriate treatment. However, there are cases in which the cause cannot be identified. This report presents a case in which the patient awoke from general anesthesia and was extubated but subsequently developed a DOC. Despite various examinations, the cause of the disorder could not be identified.

## Case presentation

The patient was a man in his 60s, with a height of 169 cm and a weight of 65.2 kg. Two months earlier, he had fallen and been diagnosed with a fracture of the left femur, for which he underwent total hip arthroplasty. Two months later, he fell again and was diagnosed with a periprosthetic fracture around the left artificial hip joint, necessitating revision total hip arthroplasty under general anesthesia. His medical history included hypertension, cerebral hemorrhage, and dyslipidemia. He was on medications, including amlodipine, olmesartan, and rosuvastatin. He also had partial left-sided paralysis due to the old cerebral hemorrhage. There was no significant family history. Preoperative electrocardiogram and blood tests showed no abnormalities.

**Figure 1:**
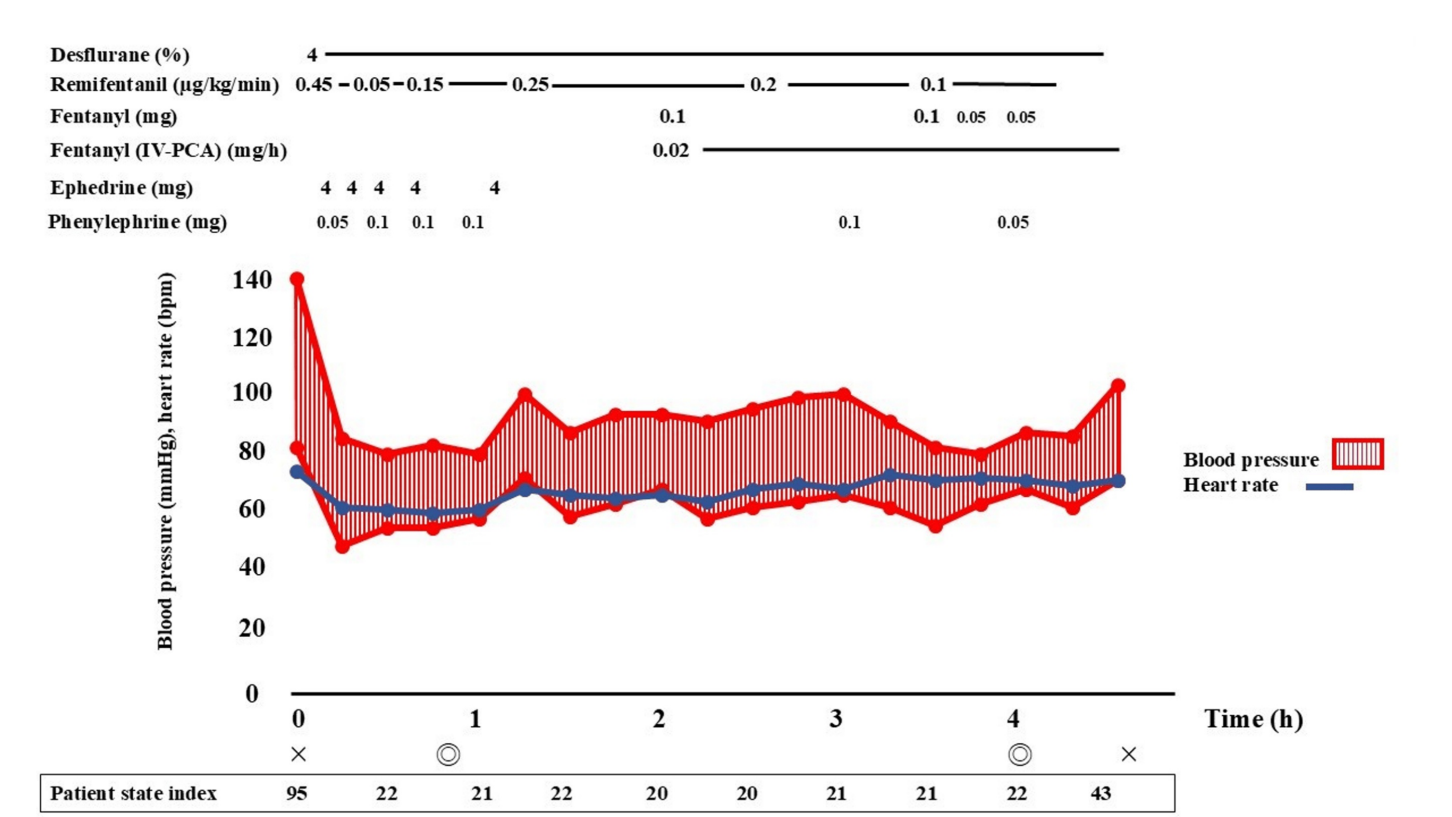
Patient’s anesthesia record, showing stable cardiovascular dynamics during surgery IV-PCA: intravenous patient-controlled analgesia

Anesthesia was induced with 60 mg of propofol, remifentanil at 0.45 µg/kg/minutes, and 40 mg of rocuronium. Maintenance was performed with inhalation of 4% desflurane, continuous administration of remifentanil at 0.3-0.5 µg/kg/minute, and intermittent administration of rocuronium (10 mg). An 8-mm endotracheal tube was used for intubation. Ephedrine and phenylephrine were administered as needed to maintain blood pressure during induction. During the procedure, circulatory dynamics and oxygenation were stable, showing no particular abnormalities (Figure 1).

Sedation was monitored using Sedline (Masimo, Irvine, CA, USA), with brainwaves continuously observed. The patient state index ranged between 20 and 40. For postoperative pain control, intravenous fentanyl and acetaminophen were administered, and 20 mL of 0.375% ropivacaine was locally applied to the surgical field. To reduce blood loss, 1,000 mg of tranexamic acid was administered before the start of surgery, followed by continuous infusion at 100 mg/hour until the end of the procedure. The surgery lasted 199 minutes, and the total anesthesia time was 267 minutes. Total blood loss was 100 mL. The surgery was completed without any issues, and the administration of anesthetics was discontinued. Once the end-tidal desflurane concentration reached 0.6%-0.7%, the patient was called by name, and eye-opening was observed. After confirming spontaneous breathing (six to eight breaths/minute) and the ability to follow commands, sugammadex was administered, and extubation was performed. As for the power in four limbs before extubation, there was no change in the preoperative state. After confirming that circulatory dynamics were stable and while preparing to transfer the patient from the operating room, glossoptosis occurred, and a decrease in oxygenation was observed (SpO_2_ of 80%-90%). As a result, a nasal airway was inserted. After inserting the airway, oxygenation improved, but the patient complained of discomfort from the airway, so it was removed, and the patient was transferred to the recovery room. Glossoptosis re-appeared upon transfer to the recovery room, and the patient’s consciousness level was recorded as E3V1M3-4 on the Glasgow Coma Scale (GCS). Considering the possibility of residual effects from the anesthetics, the patient was observed in the recovery room for 20-30 minutes. Respiratory status (8-10 breaths/minute) and circulatory dynamics showed no significant changes. A differential diagnosis of psychogenic nonepileptic seizures was considered for the decreased consciousness level, but the diagnosis could not be confirmed at this stage. Blood gas analysis was performed to search for causes of disturbance of consciousness, but pH, partial pressure of carbon dioxide, and electrolyte levels were within normal ranges, there was no hypoglycemia, and no specific findings were identified (Table 1).

**Table 1:** Blood gas analysis No abnormal values are found that could cause a DOC DOC: disorder of consciousness

Analyte	Result
pH	7.41
pO_2_ (mmHg)	120
pCO_2_ (mmHg)	42
Na^+^ (mmol/L)	140
K (mmol/L)	3.76
Ca^2+^ (mg/dL)	1.12
Glucose (mg/dL)	116

**Figure 2:**
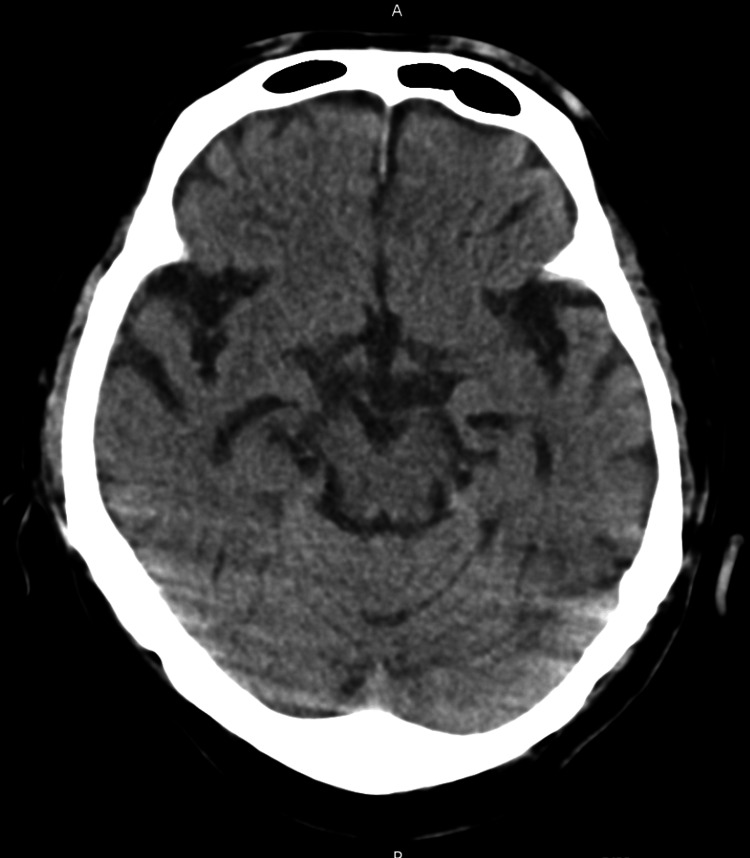
Head CT, showing no bleeding or infarct lesions CT: computed tomography

**Figure 3:**
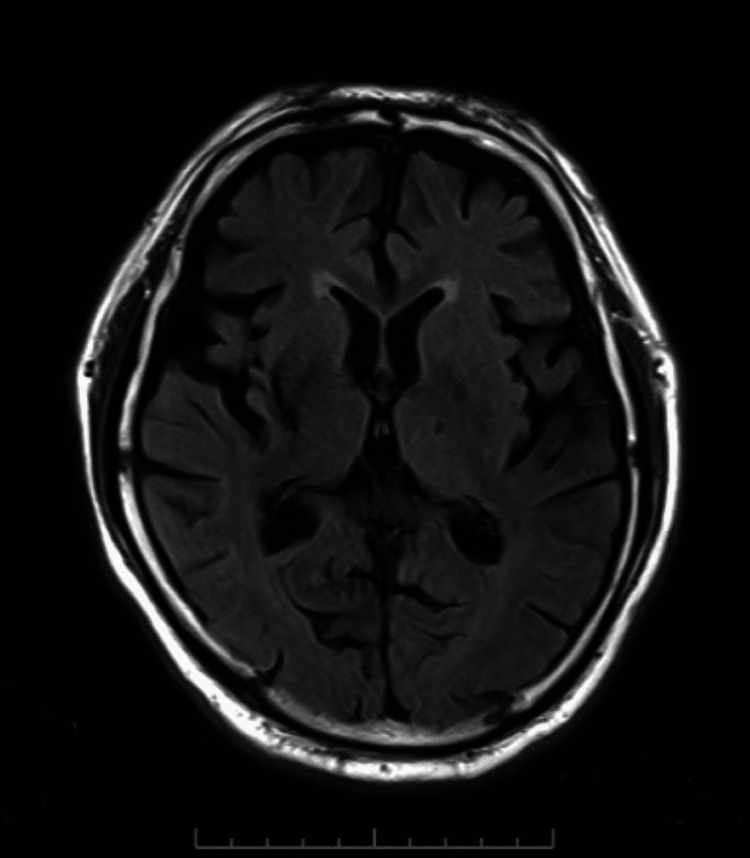
Head MRI, showing no new infarct lesions MRI: magnetic resonance imaging

During observation in the recovery room, involuntary movements of the right upper limb emerged. Head computed tomography (CT) and magnetic resonance imaging (MRI) were performed to investigate intracranial lesions, but no new brain infarction or hemorrhage was found with either test (Figures 2, 3).

Sedline brainwave monitoring was also conducted, but no seizure activity was observed. During the various tests, the consciousness level gradually improved. By 60-120 minutes after leaving the operating room, the patient’s consciousness level improved to the extent that he could engage in conversation (E4V5M6 on GCS). There were no further decreases in consciousness level, but for monitoring purposes, the patient was urgently admitted to the intensive care unit (ICU). In the ICU, no significant neurological abnormalities were observed, and the patient was discharged from the ICU the following day and subsequently discharged on the 73rd postoperative day.

## Discussion

In this case, although the patient initially awakened and was able to follow commands postoperatively, he subsequently developed an unexplained DOC, which made diagnosis challenging. DOC is the loss of consciousness. DOCs are generally caused by several types of brain damage, either individually or in combination. Regardless of the etiology, a common pathophysiological mechanism underlying coma is said to be the widespread loss of excitatory synaptic activity across the cerebral cortex [2]. Moreover, there are numerous potential causes of DOCs during the perioperative period [3,4].

In this case, the decreased consciousness level following general anesthesia could be attributed to residual effects of anesthesia, cerebrovascular events such as bleeding or ischemia, and other metabolic disorders [5]. Given the presence of involuntary movements in addition to the DOC, differential diagnoses included epileptic seizures [6]. Other potential differential diagnoses considered were tranexamic acid-related seizures due to high-dose administration and local anesthetic toxicity [7,8]. CT and MRI were performed, and no new findings of brain hemorrhage or infarction were observed. In addition to imaging, a simplified electroencephalogram was conducted, which did not show epileptiform activity. Although psychogenic nonepileptic seizures were also considered, their likelihood appeared low based on the clinical course and symptoms [9]. Reports have noted nonconvulsive status epilepticus (NCSE) in cases of perioperative DOCs [10]. NCSE can occur in patients with DOCs in whom imaging does not show the cause and can often be missed due to the absence of obvious convulsions, leading to delayed diagnosis and treatment. However, delayed treatment can potentially lead to irreversible brain damage, making early diagnosis and intervention crucial [11]. In patients with high sensitivity to opioids, there may be prolonged effects of opioids, which could also explain the DOC due to delayed effects of remifentanil or fentanyl [12]. Also, residual muscle paralysis could be the cause of glossoptosis, and raised pCO_2_ could lead to decreased levels of consciousness. Although gradual improvement in consciousness was observed during testing, a definitive diagnosis was not reached in this case. Fortunately, the patient had a good outcome without any residual effects. In cases of DOCs such as this, there is a high possibility of postoperative cognitive dysfunction, which can impact the prognosis [13]. Therefore, prompt diagnosis and intervention are deemed necessary for perioperative DOCs. Perioperative DOCs are a significant issue for anesthesiologists, requiring prompt evaluation of potential causes and implementation of appropriate treatment strategies.

## Conclusions

In this case, the patient developed a DOC after awakening from general anesthesia, and despite various tests, a definitive diagnosis was not reached. It is common for DOCs after general anesthesia to be caused by residual effects of anesthetic agents, making it challenging to identify the exact cause. Fortunately, in this case, the DOC improved without leading to severe neurological complications.
